# DORN1/P2K1 and purino-calcium signalling in plants: making waves with extracellular ATP

**DOI:** 10.1093/aob/mcz135

**Published:** 2020-01-06

**Authors:** Elsa Matthus, Jian Sun, Limin Wang, Madhura G Bhat, Amirah B Mohammad-Sidik, Katie A Wilkins, Nathalie Leblanc-Fournier, Valérie Legué, Bruno Moulia, Gary Stacey, Julia M Davies

**Affiliations:** 1 Department of Plant Sciences, University of Cambridge, Cambridge, UK; 2 Institute of Integrative Plant Biology, School of Life Science, Jiangsu Normal University, Xuzhou, China; 3 Université Clermont Auvergne, INRA, PIAF, Clermont-Ferrand, France; 4 Divisions of Plant Science and Biochemistry, University of Missouri, Columbia, MO, USA

**Keywords:** *Arabidopsis*, ATP, calcium, DORN1, P2K1, reactive oxygen species, root, wave

## Abstract

**Background and Aims:**

Extracellular ATP governs a range of plant functions, including cell viability, adaptation and cross-kingdom interactions. Key functions of extracellular ATP in leaves and roots may involve an increase in cytosolic free calcium as a second messenger (‘calcium signature’). The main aim here was to determine to what extent leaf and root calcium responses require the DORN1/P2K1 extracellular ATP receptor in *Arabidopsis thaliana*. The second aim was to test whether extracellular ATP can generate a calcium wave in the root.

**Methods:**

Leaf and root responses to extracellular ATP were reviewed for their possible links to calcium signalling and DORN1/P2K1. Leaves and roots of wild type and *dorn1* plants were tested for cytosolic calcium increase in response to ATP, using aequorin. The spatial abundance of DORN1/P2K1 in the root was estimated using green fluorescent protein. Wild type roots expressing GCaMP3 were used to determine the spatial variation of cytosolic calcium increase in response to extracellular ATP.

**Key Results:**

Leaf and root ATP-induced calcium signatures differed markedly. The leaf signature was only partially dependent on DORN1/P2K1, while the root signature was fully dependent. The distribution of DORN1/P2K1 in the root supports a key role in the generation of the apical calcium signature. Root apical and sub-apical calcium signatures may operate independently of each other but an apical calcium increase can drive a sub-apical increase, consistent with a calcium wave.

**Conclusion:**

DORN1 could underpin several calcium-related responses but it may not be the only receptor for extracellular ATP in Arabidopsis. The root has the capacity for a calcium wave, triggered by extracellular ATP at the apex.

## INTRODUCTION

ATP is the universal cellular energy currency. In plant cells, cytosolic ATP occurs at 0.5–2 mm (Gout *et al.*, 2014; De Col *et al.*, 2017). Whilst the mechanisms of ATP release into the plant cell’s extracellular space remain under investigation (Kim *et al.*, 2006; Song *et al.*, 2006; Rieder and Neuhaus, 2011; Wu *et al.*, 2011), accumulation of extracellular ATP (eATP) to nanomolar levels occurs during growth and possibly to higher levels in response to abiotic and biotic stimuli (Jeter *et al.*, 2004; Kim *et al.*, 2006; Song *et al.*, 2006; Weerasinghe *et al.*, 2009; Clark *et al.*, 2010; Dark *et al.*, 2011; Zhu *et al.*, 2017; Nizam *et al.*, 2019). Levels of eATP are controlled through ATP-hydrolysing enzymes such as nucleotidases and most possibly by apyrases, although results on the latter are contended (Wu *et al.*, 2007; Riewe *et al.*, 2008; Lim *et al.*, 2014; Massalski *et al.*, 2015; Nizam *et al.*, 2019). Damage to the plasma membrane rapidly releases large amounts of intracellular ATP into the extracellular space (Song *et al.*, 2006; Weerasinghe *et al.*, 2009; Dark *et al.*, 2011). Extracellular ATP has therefore been termed a ‘danger signal’ (Choi *et al.*, 2014*a*). Basal levels of eATP are, however, needed for optimal plant growth, with both depletion and augmentation of eATP triggering plant stress responses and too low or high a level promoting cell death (Chivasa *et al.*, 2005; Kim *et al.*, 2006; Clark *et al.*, 2010; Sun *et al.*, 2012; Jia *et al.*, 2019). Thus, eATP has the hallmarks of a tightly regulated plant cell regulator.

eATP causes accumulation of reactive oxygen species (ROS), nitric oxide (NO), Ca^2+^ and phosphatidic acid, all of which are significant plant signalling intermediates (Demidchik *et al*., 2003*a*, 2009; Tonón *et al.*, 2010; Salmi *et al.*, 2013; Clark and Roux, 2018; Q. W. Wang *et al.*, 2019*a*). These may be involved in the changes in gene expression and protein abundance upon eATP perception (Jeter *et al.*, 2004; Demidchik *et al.*, 2009; Choi *et al.*, 2014*b*; Lim *et al.*, 2014; Lang *et al.*, 2017; Tripathi *et al.*, 2018; Jewell *et al.*, 2019). Studies to date on *Arabidopsis thaliana* seedlings support enrichment of defence- and wound-response genes in the suite regulated by eATP (Choi *et al.*, 2014*b*; Tripathi *et al.*, 2018; Jewell *et al.*, 2019). The use of Arabidopsis receptor and signalling mutants has revealed that subsets of the eATP-regulated genes also require input from the jasmonate, ethylene and salicylic acid pathways (Tripathi *et al.*, 2018; Jewell *et al.*, 2019). The receptor on which eATP research currently hinges was identified through a forward genetic screen as a plasma membrane-spanning legume-like lectin serine–threonine receptor kinase termed ‘DOes not Respond to Nucleotides1’ (DORN1; Choi *et al.*, 2014*b*). The first higher plant eATP receptor, DORN1 (also known as P2K1 to align with animal purino-receptor nomenclature) has been characterized previously as LecRK1-9, which is a regulatory component of the plasma membrane–cell wall continuum and is important for pathogen resistance (Bouwmeester *et al.*, 2011; Balagué *et al.*, 2017; Tripathi *et al.*, 2018).

DORN1 came to light as an eATP receptor through its role in increasing cytosolic free Ca^2+^ ([Ca^2+^]_cyt_) in seedlings (Choi *et al.*, 2014*b*). This ability to direct [Ca^2+^]_cyt_ as a second messenger is pivotal to plant purino signalling. Elevation of [Ca^2+^]_cyt_ in plants is held to be stimulus-specific with the distinct spatio-temporal patterns of [Ca^2+^]_cyt_ elevation (‘signatures’) generated by the combined activities of plasma, and endomembrane Ca^2+^-transport proteins and Ca^2+^-binding proteins (McAinsh and Pittman, 2009). A signature can generate a specific transcriptional response, for example through Ca^2+^-dependent transcription factors such as CAMTAs (Lenzoni *et al.*, 2018). Whole seedlings of Arabidopsis generate a robust [Ca^2+^]_cyt_ signature in response to eATP that requires DORN1 (Jeter *et al.*, 2004; Choi *et al*., 2014*a*,*b*; Nizam *et al.*, 2019). Critically, the DORN1-dependent eATP-regulated transcriptome runs in part through CAMTA3 (Jewell *et al.*, 2019), linking the eATP signal to transcriptional output via [Ca^2+^]_cyt_. Further resolution of this signalling cascade requires deconstruction of the seedling’s eATP-[Ca^2+^]_cyt_ signature. Roots are known to contribute to the overall [Ca^2+^]_cyt_ signature of the seedling (Demidchik *et al*., 2003*a*, 2009, 2011; Rincón-Zachary *et al.*, 2010; Tanaka *et al.*, 2010; Loro *et al*., 2012, 2016; Costa *et al.*, 2017, Matthus *et al.*, 2019) but whether DORN1 operates in the [Ca^2+^]_cyt_ signatures of only roots or leaves as well is unknown. In this review, the breakthrough finding of DORN1 as an eATP receptor will be placed into the context and mechanisms of plant purine signalling (spanning growth, stress responses, wounding and cross kingdom interactions) with new data supporting the operation of DORN1 in both leaf and root [Ca^2+^]_cyt_ elevation. The mechanistic basis of leaf and root [Ca^2+^]_cyt_ signals will be discussed and the ability of eATP to generate systemic, rather than only local, signals through [Ca^2+^]_cyt_ increase will be explored. Connections will be made to other signalling pathways, the many ‘black boxes’ of plant purine signalling will be examined for content, and future directions for this emerging research area will be suggested.

### Leaf eATP: implications for abiotic stress and wounding

Hou *et al.* (2018) reported that hypertonic salt stress increases Arabidopsis leaf eATP and that eATP may then protect photosystem II (PSII) activity. The *dorn1-3* mutant was compromised in eATP protection of PSII (Hou *et al.*, 2018), which suggests that eATP effects on PSII may run through [Ca^2+^]_cyt_ as a second messenger. Applying gadolinium or lanthanum as blockers of plasma membrane Ca^2+^ influx channels inhibits eATP stimulation of PSII activity (Feng *et al.*, 2015), from which an eATP-induced [Ca^2+^]_cyt_ increase in leaves can be inferred. More recently, Hou *et al.* (2019) found that DORN1 is needed for the protective effects of eATP on PSII under high light. Whether eATP affects Ca^2+^ signalling in the chloroplast itself also remains to be determined. Certainly, eATP causes a plastidial Ca^2+^ increase in roots (Loro *et al.*, 2016). Additionally, in leaves DORN1 is implicated in transducing the eATP signal induced in response to cadmium stress (Hou *et al.*, 2017). This would be an interesting test case for the involvement of [Ca^2+^]_cyt_, as cadmium can interfere with Ca^2+^ transport and signalling due to its similar size (Wilkins *et al.*, 2016).

Given that eATP increases on wounding, and governs wound-induced/jasmonate-dependent transcription through DORN1 (Choi *et al.*, 2014*b*; Tripathi *et al.*, 2018; Jewell *et al.*, 2019), it is timely to consider this receptor’s position in wound-induced [Ca^2+^]_cyt_ signalling. Blocking plasma membrane Ca^2+^ influx channels impairs eATP induction of the jasmonate-dependent transcripts implicated in leaf wounding and necrotroph attack (Tripathi *et al.*, 2018), potentially putting DORN1 upstream of channel opening. Leaf wounding (including by insect feeding) triggers a local [Ca^2+^]_cyt_ elevation (Kiep *et al.*, 2015; Vincent *et al.*, 2017) and a vascular [Ca^2+^]_cyt_ ‘wave’ that signals to other leaves, evoking a distal transcriptional response (Kiep *et al.*, 2015; Nguyen *et al.*, 2018; Toyota *et al.*, 2018). The local [Ca^2+^]_cyt_ signal involves two glutamate receptor-like Ca^2+^ influx channels (GLR 3.3 and 3.6; Vincent *et al.*, 2017) and the TPC1 (two pore channel1) vacuolar Ca^2+^ release channel (Kiep *et al.*, 2015; Vincent *et al.*, 2017), while the wave is underpinned by GLR 3.3 and 3.6 (Nguyen *et al.*, 2018; Toyota *et al.*, 2018). At present, there is some doubt as to whether those GLRs operate at the plasma membrane (Toyota *et al.*, 2018) or in endomembranes (Nguyen *et al.*, 2018) in the vascular [Ca^2+^]_cyt_ wave. Termination of the wound-induced [Ca^2+^]_cyt_ signal is mediated in part by the plasma membrane ACA8 Ca^2+^-ATPase Ca^2+^ efflux pump (Costa *et al.*, 2017).

Leaf wounding causes accumulation of extracellular glutamate (Toyota *et al.*, 2018). It may be that DORN1 plays a part in the local wound [Ca^2+^]_cyt_ signal or wave, especially given the finding that glutamate can cause eATP accumulation (Song *et al.*, 2006; Dark *et al.*, 2011). This could also help to explain the occurrence of apyrase in caterpillar saliva, which can suppress plant jasmonate-dependent wounding responses (Wu *et al.*, 2012). The caterpillar apyrase could hydrolyse plant eATP to prevent a [Ca^2+^]_cyt_-driven wound response, a scenario supported by the finding that insect oral secretions suppress wound-induced [Ca^2+^]_cyt_ elevation in leaves (Kiep *et al.*, 2015). Recent work on kidney bean (*Phaseolus vulgaris*) leaves suggests that local wound-induced eATP elevation causes ROS elevation not only locally but also in other leaves, potentially through an ROS ‘wave’ (Q. W. Wang *et al.*, 2019*a*). In Arabidopsis roots, [Ca^2+^]_cyt_ and ROS work together in wave propagation for abiotic stress signalling (Evans *et al.*, 2016). High-resolution studies (using leaf cell-specific aequorin lines; Marti *et al.*, 2013) or more sensitive [Ca^2+^]_cyt_ reporters are needed to determine the spatial extent of DORN1’s operation in leaf eATP Ca^2+^ signatures and waves. Cell-specific lines would be particularly relevant to testing for DORN1’s role in guard cells.

### DORN1 and eATP operate in stomatal aperture regulation

Guard cells use [Ca^2+^]_cyt_ as a second messenger in aperture control (a system that involves plasma membrane NADPH oxidases and Ca^2+^ channels; Jezec and Blatt, 2017), and are now known to respond to eATP. Clark *et al.* (2011) reported eATP-induced production of ROS and NO with closure of Arabidopsis stomata in the light, but opening in the dark. Perhaps high light-driven ATP production results in greater eATP to close stomata and protect from evapotranspiration. Certainly, light levels control the triggering of cell death by eATP depletion in tobacco (*Nicotinia tabacum*; Chivasa *et al.*, 2009). eATP promotion of stomatal opening was found to require the heterotrimeric G protein alpha subunit (GPA1) and plasma membrane NADPH oxidases RBOHD and F, with plasma membrane Ca^2+^ influx detected (Hao *et al.*, 2012). In *Vicia faba* guard cells, plasma membrane Ca^2+^ channel activity was enhanced by eATP, which also promoted opening (Wang *et al.*, 2014).

Stomatal closure by eATP involves DORN1. *dorn1* mutants do not close in response to eATP yet can still close when challenged by abscisic acid (ABA) (Chen *et al.*, 2017), even though exogenous ABA can induce eATP accumulation by guard cells (Clark *et al.*, 2011). The RBOHD NADPH oxidase is a common component of both eATP and ABA pathways (Kwak *et al.*, 2003; Chen *et al.*, 2017). RBOHD directly interacts with DORN1 and eATP-induced stomatal closure fails in *rbohD* mutants (Chen *et al.*, 2017). A recent transcriptional study of the *rhbohd* mutant under high light stress found that some eATP-dependent transcripts were misregulated, also indicating that eATP signalling may run through this NADPH oxidase (Zandalinas *et al.*, 2019). The DORN1 pathway is also proposed to operate in stomatal closure in response to pathogen attack (Chen *et al.*, 2017). Whether DORN1 can contribute to the guard cell [Ca^2+^]_cyt_ oscillations that occur in defence signalling (Thor *et al.*, 2014) could be tested using ratiometric fluorescence imaging of [Ca^2+^]_cyt_.

Clearly, understanding how eATP can induce both stomatal opening and closure (potentially through RBOHD) requires further investigation, with the premise of dose-dependency (Clark *et al.*, 2011) as a logical entry point. The mechanism of release of ATP to the extracellular space is central to further work in this area. Guard cell eATP accumulation is impaired in Arabidopsis mutants lacking the MRP4/5 ABC transporters (Wang *et al.*, 2015). These have been proposed to be at the plasma membrane, suggesting that they mediate efflux of ATP (Wang *et al.*, 2015). This could in turn relate to the impaired guard cell plasma membrane Ca^2+^ channel activity of the *mrp5* mutant (Suh *et al.*, 2007), with the possibility that the channel lesion is due to lower eATP levels leading to a failure in channel activation. However, a green fluorescent protein (GFP) localization study using MRP5’s native promoter (rather than constitutive expression) revealed its vacuolar origin, and heterologous expression showed MRP5 to be an inositol hexa*kis*phosphate (IP_6_) transporter (Nagy *et al.*, 2009). As IP_6_ regulates endomembrane Ca^2+^ release (Lemtiri-Chlieh *et al.*, 2003) and plasma membrane K^+^ influx (Lemtiri-Chlieh *et al.*, 2000), a more complicated picture of guard cell eATP accumulation emerges that potentially involves inositol signalling and with a black box still at the plasma membrane for ATP efflux.

### eATP at work in roots: growth and navigation

Ratiometric imaging has revealed that cytosolic ATP (as the MgATP^2−^ species) is heterogeneously distributed through the Arabidopsis root (de Col *et al.*, 2017). This is true also for eATP, with greatest levels at the root cap and root cell expansion points, particularly the root hair apex (Kim *et al.*, 2006; Weerasinghe *et al.*, 2009). High concentrations of exogenous (experimentally applied) ATP inhibit root elongation, probably through elevation of auxin, disruption of vesicular trafficking and increased cell wall lignification (Tang *et al.*, 2003; Liu *et al.*, 2012; Lim *et al.*, 2014; Deng *et al.*, 2015; Yang *et al.*, 2015; Zhu *et al.*, 2018). Prolonged exposure to high exogenous ATP (0.8 mm for 12 h) causes root cell death (Deng *et al.*, 2015), so *in planta* eATP must be tightly regulated. Downregulation of apoplastic apyrase (which could elevate eATP) increases potato (*Solanum tuberosum*) tuber number and alters their shape (Riewe *et al.*, 2008). eATP concentration appears critical to growth regulation of root hairs, with 7.25–25 µm exogenously added ATP or ADP stimulating elongation but ≥150 µm inhibiting elongation (Clark *et al.*, 2010; Terrile *et al.*, 2010). It is tempting to speculate that the low cytosolic MgATP^2−^ that correlates with high root hair growth rate (de Col *et al.*, 2017) reflects release of ATP to the apex to drive elongation. Chelation of extracellular Ca^2+^ or application of plasma membrane Ca^2+^ channel inhibitors lowers root cell eATP levels and indicates a reliance on Ca^2+^ influx (Kim *et al.*, 2006). It may be that plasma membrane mechanosensitive Ca^2+^-permeable channels are responsible for the influx, opening in response to membrane stretch during cell expansion. As increased [Ca^2+^]_cyt_ can stimulate exocytosis (Carroll *et al.*, 1998), release of ATP as possible cargo of exocytotic vesicles (Yang *et al.*, 2015) would help explain why root cell eATP levels are lowered by the exocytosis inhibitor brefeldin A (Kim *et al.*, 2006). The growth effects of eATP on root hairs involve the production of NO and ROS, with RBOHD and F implicated in the latter (Clark *et al.*, 2010). Whether DORN1 is the root hair eATP receptor remains unknown. Neither is it clear yet whether extracellular purine nucleotides affect root hair [Ca^2+^]_cyt_, even though the latter is an important component of elongation and is increased by ROS (Foreman *et al.*, 2003). Lew and Dearnaley (2000) found that eATP and eADP could depolarize the plasma membrane potential of Arabidopsis root hairs, which would be consistent with Ca^2+^ influx, but when testing eADP found no effect on [Ca^2+^]_cyt_.

Touch causes a transient, asymmetric increase in root eATP (Weerasinghe *et al.*, 2009; Dark *et al.*, 2011). Subjecting Arabidopsis roots to a mechanical stress typical of that experienced on growth through soil leads to increased concentrations of eATP, approximately 60 nm at 1 min after application (Weerasinghe *et al.*, 2009), with higher levels on the side touched. The distal elongation zone was the most responsive root part, with both heterotrimeric G protein alpha (GPA1) and beta subunits (AGB1) found to be necessary for limiting the refractory period of eATP accumulation on repeated stimulation (Weerasinghe *et al.*, 2009). As mechanical stress increases root [Ca^2+^]_cyt_ (Legué *et al.*, 1997), it is again reasonable to invoke the involvement of plasma membrane mechanosensitive Ca^2+^ channels as operating upstream of eATP release. Alternatively, as members of the MSL family of mechansosensitive channels are anion-permeable (Basu and Haswell, 2017), perhaps they underpin the efflux of anionic ATP species. The site of eATP accumulation and local concentration may well help to direct the root’s growth response after contact with an obstacle. The Arabidopsis *gpa1agb1* mutant is impaired in touch-induced obstacle avoidance (Weerasinghe *et al.*, 2009), implying the need for tight control of eATP in mechanosensing. DORN1 has yet to be placed in this phenomenon.

Mechanosensing also has a place in root skewing and waving, which occurs when roots grow on a vertical or tilted surface and involves contact of the root apex with the substratum (Yang *et al.*, 2015). Exogenous ATP enhances root skewing of Arabidopsis and this requires the activity of the predominant plasma membrane H^+^-ATPase, AHA2 (Tang *et al.*, 2003; Haruta and Sussman, 2012; Yang *et al.*, 2015). Skewing can also be increased by raising eATP through mutation of the apyrases that would normally hydrolyse ATP to regulate its levels (Yang *et al.*, 2015). Antagonists of animal purinoceptors prevent skewing by exogenous ATP (Yang *et al.*, 2015) but there are as yet no reports for the involvement of the plant purinoceptor, DORN1. A root will also grow away from high exogenous ATP, supporting the premise that eATP distribution and dose *in planta* are critical to growth regulation. This avoidance response requires extracellular Ca^2+^ (implying Ca^2+^ influx) and GPA1 to effect an asymmetric distribution of the PIN2 auxin transporter and accumulation of inhibitory levels of auxin at the opposite side of the root to the eATP (Zhu *et al.*, 2018). This results in the root ‘bending’ away from the eATP and occurs independently of DORN1 (Zhu *et al.*, 2018), implying the existence of other eATP receptors.

### eATP at work in roots: symbiosis, mutualism and adaptation

Few studies have addressed whether eATP operates in root symbioses. Tanaka *et al.* (2015) proposed that legumes contain a specific type of extracellular apyrase. In soybean (*Glycine max*), silencing of the GS52 extracellular apyrase suppressed nodule development and maturation (Govindarajulu *et al.*, 2009). This could be offset by the application of ADP as the product of apyrase activity. The *Dolichos biflorus* LNP root hair extracellular apyrase binds to the *Rhizobium* lipo-chitin Nod factor and inhibiting the activity of this apyrase impairs both root hair deformation and nodulation (Kalsi and Etzler, 2000). As eATP accumulates at the apex of *Medicago truncatula* root hairs (Kim *et al.*, 2006), this suggests that eATP concentrations at the root hair apex must be lowered (or those of eADP increased) to allow symbiotic signalling. Sensing the presence of fungi could involve eATP. A fungal polysaccharide extract caused eATP accumulation by *Salvia miltiorrhiza* hairy root culture (Wu *et al.*, 2011). In that study, eATP accumulation was prevented by anion channel antagonists, implicating efflux through plasma membrane anion channels. Whether symbionts (or pathogens) export ATP or ADP to communicate with or confound the plant root will be a challenging but interesting line of enquiry. Very little is known of eATP in fungal biology (Cai, 2012). For example, inhibiting the activity of ecto-nucleotidases of the rice blast fungus *Magnaporthe oryzae* inhibited germination of conidia and appresorium formation, implicating eATP as a regulator (Long *et al.*, 2015). Recently, eATP levels of Arabidopsis and barley (*Hordeum vulgare*) roots were found to increase in the early (biotrophic) phase of colonization by the endophytic fungus *Serendipita indica* (formerly known as *Piriformospora indica*) (Nizam *et al.*, 2019). The fungus secretes an ecto-5′-nucleotidase with which to lower eATP levels and permit greater colonization. The inability to sense eATP in the Arabidopsis *dorn1-3* mutant also allows extensive colonization (Nizam *et al.*, 2019). eATP activates conidiation in *Trichoderma viride*; conidiation is a wound response and intriguingly can be mimicked by eATP, acting upstream of [Ca^2+^]_cyt_ elevation (Medina-Castellanos *et al*., 2014, 2018). eATP as a wound signal therefore appears ancient and conserved.

Wounding of roots can be envisaged to occur through herbivory, and indeed mechanical wounding of Arabidopsis roots causes a transient increase in eATP (Dark *et al.*, 2011). As extracellular glutamate can also induce eATP accumulation by Arabidopsis roots (Dark *et al.*, 2011), there may be parallels to be drawn with the herbivore-induced GLR-eATP-DORN1 signalling hypothesized in a previous section. Experimentally, it will be important in further exploration to mimic herbivore-induced wounding of roots accurately to deduce membrane-based signalling. Gross mechanical damage of a root is not appropriate as the resultant ionic fluxes (including of Ca^2+^) are insensitive to channel blockers, indicating catastrophic membrane damage (Meyer and Weisenseel, 1997).

Abiotic stresses are good stimuli of eATP accumulation by roots. Copper stress causes eATP accumulation by wheat (*Triticum aestivum*) roots and protects against cell death (Jia *et al.*, 2019). Cold, salt and hyperosmotic stress all cause transient increases in Arabidopsis root eATP, as does ABA which would imply the involvement of eATP in acute and longer term stress responses (Dark *et al.*, 2011; Deng *et al.*, 2015). Additionally, salt stress increases eATP of *Glycyrrhiza uralensis* (licorice) roots (Lang *et al.*, 2014). For salt stress, eATP can play a positive role in Na^+^/K^+^ homeostasis in *Populus euphratica* suspension cells (Sun *et al.*, 2012) and also for mangrove and licorice roots (Lang *et al*., 2014, 2017). Cold, salt, hyperosmotic stress and ABA all cause elevation of [Ca^2+^]_cyt_ in Arabidopsis roots as a second messenger in adaption (Wilkins *et al.*, 2016). Could eATP be a part of this? Salt-induced [Ca^2+^]_cyt_ elevation in *dorn1* seedlings did not differ from that in the wild type, leading to the conclusion that this receptor had no part to play (Choi *et al.*, 2014*b*). However, these measurements were made with aequorin and would be the average of a variety of cell populations, potentially masking root cell-specific responses. In the next sections, the evidence for eATP elevation of root cell [Ca^2+^]_cyt_ will be reviewed and DORN1 hypothetically placed in that context.

eATP increases Ca^2+^ in specific root regions and multiple compartments of Arabidopsis root cells

The first demonstration of [Ca^2+^]_cyt_ increase by extracellular purine nucleotides was in excised Arabidopsis roots, using aequorin (Demidchik *et al.*, 2003*a*). Since then, exogenous ATP has come to be used as a standard stimulus to test the efficacy of more powerful genetically encoded [Ca^2+^]_cyt_ indicators in roots and the resolution of different imaging methods (Rincón-Zachary *et al.*, 2010; Krebs *et al.*, 2012; Loro *et al*., 2012, 2016; Bonza *et al.*, 2013; Waadt *et al.*, 2017; Kelner *et al.*, 2018). Such reporters have resolved spatially distinct regions of [Ca^2+^]_cyt_ elevation in Arabidopsis roots that are superfused experimentally with ATP (Rincón-Zachary *et al.*, 2010; Loro *et al.*, 2016; Waadt *et al.*, 2017; Matthus *et al.*, 2019). The initial [Ca^2+^]_cyt_ response occurs at the apex, with a significant contribution by the lateral root cap and meristem, followed by sub-apical elevation (Rincón-Zachary *et al.*, 2010; Tanaka *et al.*, 2010; Shi *et al.*, 2015; Behera *et al.*, 2018; Matthus *et al.*, 2019). With two spatially and temporally distinct [Ca^2+^]_cyt_ elevations evident at the root apex, the question becomes ‘does DORN1 underpin them both?’. Using such reporters, non-synchronized single-cell [Ca^2+^]_cyt_ oscillations have been found to underlie the ‘averaged’ signal that aequorin generates (Tanaka *et al.*, 2010; Krebs *et al.*, 2012; Costa *et al.*, 2017). Yellow Cameleon 3.6 (YC3.6) targeted to the plasma membrane revealed differing [Ca^2+^]_cyt_ increases within a single cell, suggesting hotspots of local [Ca^2+^]_cyt_ maxima (Krebs *et al.*, 2012).

Targeting reporters to various subcellular compartments has also uncovered links between cytosolic and organellar Ca^2+^ dynamics upon eATP perception. Targeting YC3.6 to the nucleus revealed non-synchronous oscillations in nuclear-free [Ca^2+^] in response to eATP that lagged behind the [Ca^2+^]_cyt_ response (measured in different plants) by 7 min (Krebs *et al.*, 2012), while another nuclear targeted YC3.6 reported much faster Ca^2+^ increases in response to eATP (Loro *et al.*, 2012). However, dual targeting of GECO reporters to both the nucleus and the cytoplasm enabling simultaneous measurements from both compartments revealed only a [Ca^2+^]_cyt_ response to eATP in Arabidopsis root elongation zone cells (Kelner *et al.*, 2018).

eATP treatment has also prompted [Ca^2+^] increases in root mitochondria, plastids and endoplasmic reticulum (ER) (Loro *et al*., 2012, 2016; Bonza *et al.*, 2013). Mitochondrial, plastid and ER increases in [Ca^2+^] were found to be strictly related to increases of [Ca^2+^]_cyt_, i.e. the larger the [Ca^2+^]_cyt_ increase, the larger the mitochondrial/plastid/ER increase (Loro *et al*., 2012, 2016; Bonza *et al.*, 2013). Recovery of mitochondrial Ca^2+^ levels back to pre-stimulus values was much slower (more than 20 min) than the cytosol, ER and plastids (Loro *et al*., 2012, 2016; Bonza *et al.*, 2013). Therefore, each compartment has its own Ca^2+^ signature, implying signal specificity and distinct operations of Ca^2+^ transporters and binding proteins. So far, it has been found that an increase in mitochondrial free Ca^2+^ is regulated by the MICU Ca^2+^ transporter (Wagner *et al.*, 2015). The coordination of these various signatures downstream of eATP perception promises to be a fascinating area for future research.

At the mechanistic level, antagonists of plasma membrane Ca^2+^ influx channels or chelation of extracellular Ca^2+^ abolishes almost all of the eATP- or eADP-induced elevation of Arabidopsis root [Ca^2+^]_cyt_ (Demidchik *et al*., 2003*a*, 2009, 2011; Loro *et al.*, 2016; Behera *et al.*, 2018). This implies that the apoplast is the predominant source of Ca^2+^, but it does not rule out any downstream involvement of intracellular Ca^2+^ stores which might rely on an initial trigger of apoplastic Ca^2+^. Inhibition of intracellular phospholipase C signalling was shown to affect only the later stages of [Ca^2+^]_cyt_ signalling in response to eATP, leading the authors to conclude that both intracellular and apoplastic Ca^2+^ stores were involved in generating the later signal (Tanaka *et al.*, 2010). As none of the organelle-targeted Ca^2+^ reporters showed a decrease of [Ca^2+^] upon onset of the [Ca^2+^]_cyt_ response, it was reasoned that the organelles examined so far do not function as the Ca^2+^ source for the observed initial [Ca^2+^]_cyt_ increases (e.g. Bonza *et al.*, 2013). However, the involvement of other organelles such as the vacuole and Golgi in roots remains to be tested. It is noteworthy that salt-stress-induced vacuolar Ca^2+^ release in *Populus euphratica* suspension cells requires eATP and Ca^2+^ influx across the plasma membrane (Zhang *et al.*, 2015). If the root vacuole were involved, then TPC1 would be a likely candidate for mediating Ca^2+^ efflux to the cytosol as its cytosol-facing EF hands would respond to increased [Ca^2+^]_cyt_ to enable Ca^2+^-induced Ca^2+^ release. Alternatively, the vacuole could be responding to cyclical ADPR (adenosine diphosphate-ribose). This is known to promote Ca^2+^ efflux from the guard cell vacuole (Leckie *et al.*, 1998) and is synthesized in response to NO increase (Abdul-Awal *et al.*, 2016). Root NO can increase in response to eATP and may rely on Ca^2+^ influx across the plasma membrane (Wu and Wu, 2008; Clark *et al.*, 2010).

### Signalling components at the root plasma membrane

Patch clamp electrophysiology has shown that both eATP and eADP can stimulate hyperpolarization-activated Ca^2+^ channels (HACCs) in the plasma membrane of Arabidopsis mature epidermal cells that could mediate the [Ca^2+^]_cyt_ increase (Demidchik *et al*., 2009, 2011; Shang *et al.*, 2009). These results are supported by studies using extracellular ion-selective microelectrodes, which have shown net Ca^2+^ influx to the root epidermis induced by eATP and eADP (Demidchik *et al*., 2009, 2011). Root plasma membrane HACC activation by eATP also requires the heterotrimeric G protein alpha subunit (Zhu *et al.*, 2018). A more recent patch clamp analysis demonstrated that DORN1 is required for eATP activation of a root elongation zone plasma membrane channel conductance that could permit Ca^2+^ influx to the cytosol at hyperpolarized membrane voltage (Wang *et al.*, 2018). The need for a hyperpolarized (very negative) membrane voltage to permit eATP-activated Ca^2+^ influx is supported by a study on seedlings of the Arabidopsis *aha2* mutant, expressing aequorin. As mentioned previously, AHA2 is the root’s predominant plasma membrane H^+^-ATPase that generates the majority of the hyperpolarized membrane potential. AHA2’s absence caused significant diminution of the eATP-induced [Ca^2+^]_cyt_ response (Haruta and Sussman, 2012).

eATP rapidly triggers production of both intra- and extracellular ROS in roots (Kim *et al.*, 2006; Demidchik *et al*., 2009, 2011). Blocking plasma membrane Ca^2+^ channels with Gd^3+^ can effectively abolish eATP-induced [Ca^2+^]_cyt_ increase in Arabidopsis roots but only inhibits intracellular ROS accumulation by approximately 50 % (Demidchik *et al*., 2003*a*, 2009). This implies that plasma membrane Ca^2+^ channel activity lies downstream of DORN1 and is required for a significant proportion of the ROS produced. The interaction of DORN1 with RBOHD (Chen *et al.*, 2017) may yet help to explain the involvement of ROS in the root eATP-induced [Ca^2+^]_cyt_ signature. To date, only the RBOHC isoform is implicated experimentally for roots and some intracellular ROS accumulation was still observed in response to eATP in roots of the Arabidopsis *rbohc* mutant (Demidchik *et al.*, 2009), potentially pointing to the activation of other isoforms. Certainly, RBOH D and F are expressed in roots and operate in Ca^2+^-based ABA and salt stress signalling (Wilkins *et al.*, 2016).

Modelling studies suggest that the ‘decay time’ of a calcium signature plays an important role in the regulation of gene expression (Lenzoni *et al.*, 2018). However, very little is known mechanistically about how the eATP-induced [Ca^2+^]_cyt_ increase might be limited or terminated. The heterotrimeric G protein beta subunit AGB1 negatively regulates eATP-induced [Ca^2+^]_cyt_ increase in Arabidopsis seedlings, but its role in roots is unknown (Tanaka *et al.*, 2010). In contrast, the plasma membrane Ca^2+^-ATPase exporters ACA8 and ACA10 have been shown to play a role in ending the [Ca^2+^]_cyt_ signal, through Ca^2+^ imaging of mutant roots (Costa *et al.*, 2017; Behera *et al.*, 2018). Whether post-translational modification of DORN1 or its retrieval from the plasma membrane plays a part remains unknown.

### The eADP conundrum and DORN1-independent channel activation

A conundrum evident in the literature is that eADP can evoke extracellular superoxide anion production by roots, but unlike eATP it cannot induce intracellular ROS accumulation (Kim *et al.*, 2006; Demidchik *et al*., 2009, 2011). The inability of eADP to evoke intracellular ROS accumulation has also been noted in leaves (Song *et al.*, 2006). eADP and eATP are not always equivalent in effect. eADP has also been found to fail in inhibiting endocytosis (Deng *et al.*, 2015). It can, however, promote nodule formation in soybean roots whereas eATP does not (Govindarajulu *et al.*, 2009). For ROS, it has been suggested previously that eADP may somehow prevent entry of extracellular H_2_O_2_ into the cytosol (Wang *et al.*, 2018). At fine resolution, differences appear between eADP and eATP in their relationship with root [Ca^2+^]_cyt_ increase. Protoplasts from Arabidopsis mature epidermis expressing aequorin show an eADP-induced [Ca^2+^]_cyt_ increase that is resistant to the reductant dithiothreitol (Dark *et al.*, 2011). This suggests that, in contrast to eATP, there is no oxidation-dependent component. Furthermore, the eATP-activated [Ca^2+^]_cyt_ increase in such protoplasts is enhanced by neutral extracellular pH but this does not occur with eADP (Demidchik *et al.*, 2011). Patch clamping has shown that eADP can activate HACC activity in Arabidopsis plasma membrane from the mature epidermis, independently of G protein activity (Demidchik *et al.*, 2009). This appears to be in contrast to the GPA1-dependent eATP-activated HACC reported by Zhu *et al.* (2018). Finally, in patch clamp trials, the DORN1-dependent eATP-activated plasma membrane Ca^2+^ influx pathway in the elongation zone did not respond to eADP, even at a concentration orders of magnitude above DORN1’s *K*_d_ (Wang *et al.*, 2018). This begs the question of whether there is a DORN1-independent eADP pathway in some epidermal cells. There is no a priori argument against a cell’s having more than one type of purinoreceptor. As described earlier, the Arabidopsis root’s eATP avoidance response is independent of DORN1 (Zhu *et al.*, 2018). Indeed, given the evidence for multiple purinoreceptors in mammals, it might be considered surprising if plants do not also contain multiple eATP/eADP receptors. Further patch clamp analysis at the single channel rather than population level is now needed, combined with high-resolution single cell imaging of DORN1 mutants.

### Interplay of eATP with other signals

In animals, eATP can modulate other signalling pathways by interacting with their receptors (Kim *et al.*, 2018). Recently, ATP has been identified as a hydrotrope, capable of maintaining protein solubility and preventing protein aggregates (Patel *et al.*, 2017). Whether it operates in this way outside of the plant plasma membrane is an interesting possibility. As such, the interplay between eATP and other regulators in plants is an understudied area of interest. eATP can still increase root [Ca^2+^]_cyt_ after [Ca^2+^]_cyt_ has been previously elevated by auxin or glutamate (Costa *et al.*, 2013; Waadt *et al.*, 2017; Behera *et al.*, 2018) but it is not clear whether such pre-exposure to these regulators has an effect on the eATP Ca^2+^ signature or requires DORN1. In sensory synapses, eATP can cause glutamate release through purinergic receptor activation (Gu and MacDermott, 1997) and while glutamate can cause eATP accumulation in plants (Dark *et al.*, 2011), the reverse case has not been reported. This could have consequences for our understanding of glutamate-based regulation of wound signalling or other processes such as carbon/nitrogen balance and root development (Toyota *et al.*, 2018; Wudick *et al.*, 2018). eATP has been found to modulate animal N-methyl-d-aspartate (NMDA)-type glutamate receptors, acting as a competitive antagonist of glutamate binding and a positive modulator at a separate allosteric site (Kloda *et al.*, 2004). Plant GLRs have significant structural homology with NMDA receptors (Wudick *et al.*, 2018), and it will be interesting to see whether eATP can influence their activity. There may also be an intricate relationship between eATP, salicylic acid and ethylene given that treating Arabidopsis roots with ethylene weakens the eATP-induced [Ca^2+^]_cyt_ elevation (Waadt *et al.*, 2017). This may prove relevant to the coordination of growth and immune responses and it will be interesting to see whether ethylene lowers DORN1 abundance.

### Testing DORN1’s involvement and the possibility of an eATP ‘wave’

This review of extracellular purines and [Ca^2+^]_cyt_ in leaves and roots provides a *prima facie* case for DORN1’s underpinning eATP- and eADP-induced [Ca^2+^]_cyt_ in a variety of processes, but has also highlighted instances where DORN1 may be redundant. A critical first step in understanding the extent of DORN1’s involvement is to move from whole seedling studies to leaves and roots. To address this, aequorin-expressing seedlings of wild type Arabidopsis and its *dorn1-1* mutant have been dissected. DORN1’s abundance at the Arabidopsis root apex has been examined through GFP as a first test of whether it is present in root regions that undergo spatially discrete eATP-induced [Ca^2+^]_cyt_ elevations. Finally, the relationship between the two spatially distinct eATP-induced [Ca^2+^]_cyt_ elevations at the root apex have been examined to test whether they are independent of each other and could form the basis of an eATP-induced [Ca^2+^]_cyt_ ‘wave’.

## MATERIALS AND METHODS

### Plant materials and growth conditions

Arabidopsis Col-0, *dorn1-1*and *dorn1-3* constitutively expressing cytosolic (apo)aequorin were as described by Choi *et al.* (2014*b*) and the DORN1-GFP line was as described by Choi *et al.* (2014*a*). Arabidopsis expressing cytosolic GCaMP3 was as described by Vincent *et al.* (2017). Plants were grown on half-strength MS medium (Duchefa), solidified with 0.8 % (w/v) bactoagar (BD Biosciences), as described by Matthus *et al.* (2019).

### Measurement of [Ca^2+^]_cyt_ with aequorin

An individual leaf or root from seedlings constitutively expressing cytosolic (apo)aequorin was treated with 10 µm coelenterazine (NanoLight Technology) as described by Matthus *et al.* (2019) with the exception that roots were bathed in 10 mm CaCl_2_, 0.1 mm KCl, 2 mm MES/Tris, pH 5.8. Recordings of luminescence from individual leaves or roots were made with a plate reader as described by Matthus *et al.* (2019). Control solution for leaves was liquid half-strength MS medium with 1.175 mm MES, adjusted to pH 5.6 using Tris. This was used for roots by Matthus *et al.* (2019), allowing some comparison. Control solution for roots was 10 mm CaCl_2_, 0.1 mm KCl, 2 mm MES/Tris, pH 5.8 (Laohavisit *et al.*, 2013). Test treatments were with adenosine 5′-triphosphate disodium salt trihydrate (ATP; Melford) or adenosine 5′-diphosphate disodium salt dehydrate (ADP; Melford) dissolved in control solution and buffered back to the original pH. Estimation of [Ca^2+^]_cyt_ was as described by Matthus *et al.* (2019).

### Measurement of [Ca^2+^]_cyt_ with GCaMP3 and DORN1 abundance with GFP

A 10-d-old Arabidopsis Col-0 seedling (expressing cytosolic GCaMP3; Vincent *et al.*, 2017) was placed across a gap in the growth medium agar. Twenty seconds after the start of image acquisition, 3 µL of control or 1 mm ATP solution was applied to the root tip. Then at 295 s, 3 µL of control or 1 mm ATP solution was applied to the mature zone. In a separate set of experiments, the application was reversed, with eATP's being added to the mature zone first then the root tip. Images were taken for 495 s in total. Images were captured with a Leica M205 FA stereo microscope, with a DFC365FX camera (Leica) and a Sola SE365 light source (Lumencor), which allowed excitation at 470/40 nm and using a GFP-ET filter collected emission at 525/50 nm, with a 500 ms exposure time, a gain of 2.0 and 30× magnification. The software LAS X (Leica) was used to control the microscope, light source and camera. ImageJ Fiji was used to process the GCaMP3 GFP signal intensities. Z-axis profiles were plotted for each region of interest, and background signal was subtracted. The protocol for normalizing GFP fluorescence (Δ*F*/*F*_0_) was taken from Vincent *et al.* (2017). For DORN1-GFP determination, root tips were imaged on a Leica SP5 DM6000B confocal microscope, using a 20× objective (HC PL APO 20×/0.7). GFP was excited at 488 nm using an argon laser, set to 30 % laser power. GFP fluorescence emission was measured at 500–540 nm. Throughout the experiments the settings were kept constant (pinhole: 221.91 µm, gain set to 600 and speed of scanning to 400 Hz). Brightfield images were also taken of every imaged root section.

## RESULTS

### Leaf eATP-dependent [Ca^2+^]_cyt_ elevation requires DORN1

**Fig. 1.**
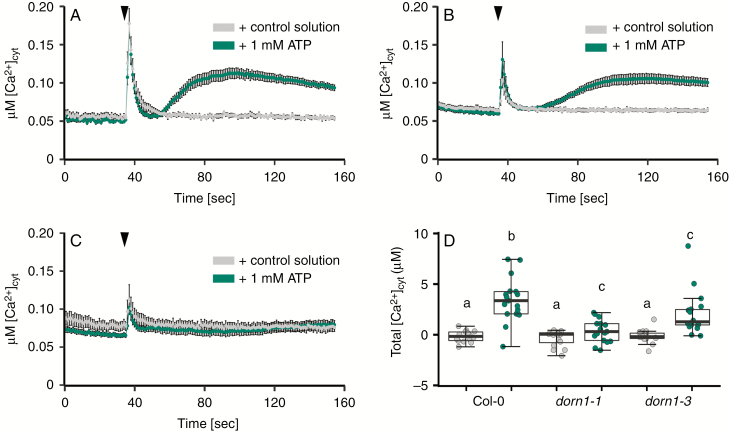
DORN1 governs the leaf eATP-induced [Ca^2+^]_cyt_ increase. (A) Mean ± s.e.m. [Ca^2+^]_cyt_ time course of individual excised leaves from 11-d-old *Arabidopsis thaliana* Col-0 constitutively expressing cytosolic aequorin. Plants were grown on solidified half-strength MS medium then leaves were assayed in liquid medium as described by Matthus *et al.* (2019). Control medium or with 1 mm ATP (pH 5.6) was added at 35 s (black triangle); *n* = 15–19 leaves in three independent trials. (B) As (A) but with 14-d-old Col-0 leaves, at pH 5.8. (C) Response of 14-d-old *dorn1-1* leaves. (D) Total [Ca^2+^]_cyt_ mobilized (estimated as the area under the curve, after subtraction of mean pre-stimulus baseline) in response to control medium (grey points) or 1 mm ATP (green points) by 14-d-old Col-0, *dorn1-1* and *dorn1-3* leaves; Col-0 *n* = 14–17, *dorn1-1 n* = 16–17, *dorn1-3 n* = 18 in three independent trials. Each dot represents an individual data point, and the thick black line denotes the median. Different lower-case letters describe groups with significant statistical difference (*P* ≤ 0.05), while data points with the same letter indicate no statistical significance (*P* ≥ 0.05; Welch’s two sample *t*-test).

The Arabidopsis leaf eATP [Ca^2+^]_cyt_ signature has not been shown in the literature. Here, addition of control solution caused a brief monophasic [Ca^2+^]_cyt_ increase due to mechanical disturbance (‘touch response’) in 11-d-old and 14-d-old Col-0 leaves (Fig. 1A, B). In contrast, eATP caused a sustained [Ca^2+^]_cyt_ increase in Col-0, regardless of plant age (Fig. 1A, B). This [Ca^2+^]_cyt_ signature had a markedly different time course and lower magnitude than the typically biphasic root [Ca^2+^]_cyt_ response (Demidchik *et al.*, 2003*a*; Matthus *et al.*, 2019). Baseline [Ca^2+^]_cyt_ was not recovered during the recording. The whole seedling eATP-induced [Ca^2+^]_cyt_ increase appears to be entirely dependent on DORN1, as loss of function mutants do not respond (Choi *et al.*, 2014*b*). Results here show that this is not as clear cut for leaves. Those of the *dorn1-1* kinase-domain mutant (Fig. 1B, D) and the *dorn1-3* ATP-binding-domain mutant (Fig. 1D) still supported a significant eATP-induced [Ca^2+^]_cyt_ increase, but not to the same level as the Col-0 wild type. At the level of resolution afforded by aequorin, DORN1 therefore appears important to the leaf’s eATP-dependent [Ca^2+^]_cyt_ response but is potentially partially redundant.

The root eATP-induced and eADP-induced [Ca^2+^]_cyt_ signatures are DORN1-dependent

**Fig. 2.**
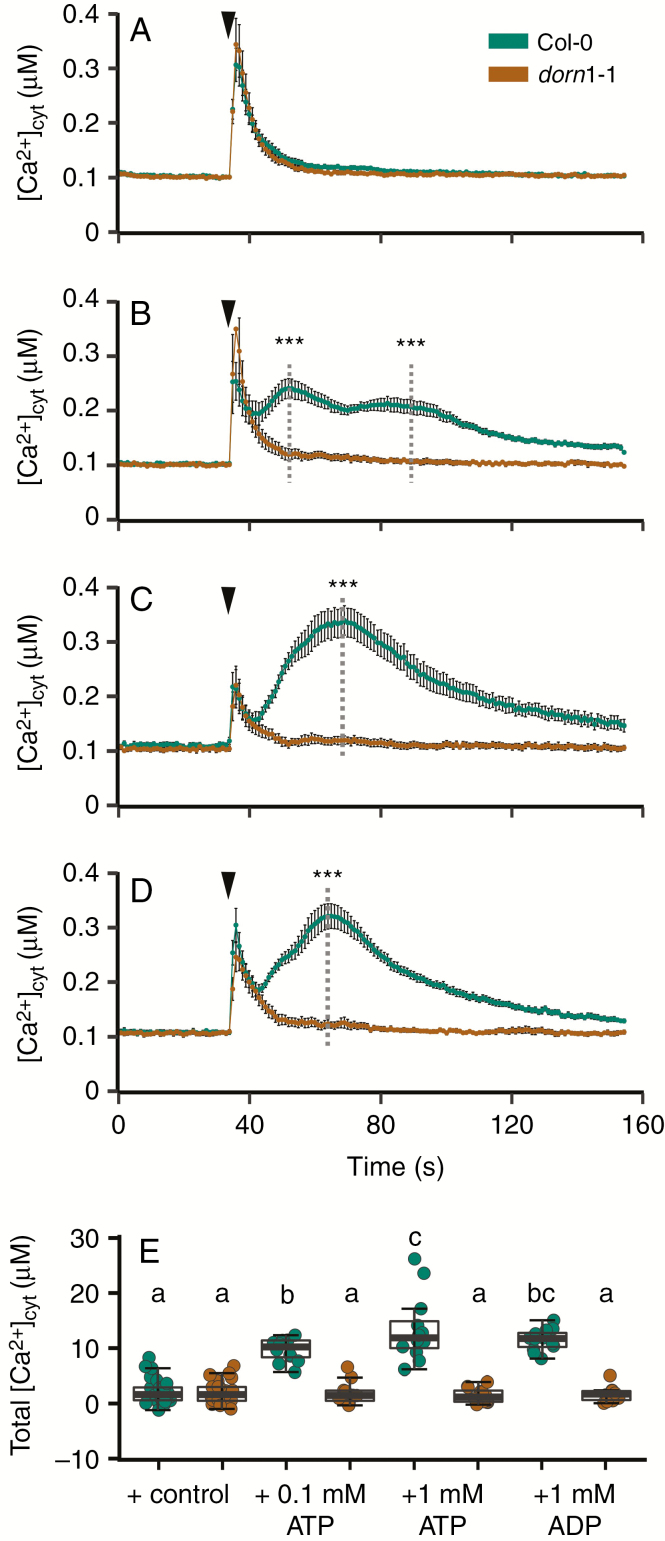
DORN1 governs the root eATP-induced and eADP-induced [Ca^2+^]_cyt_ increase. (A) Mean ± s.e.m. [Ca^2+^]_cyt_ time course of individual excised roots from 7-d-old *Arabidopsis thaliana* Col-0 and *dorn1-1* constitutively expressing cytosolic aequorin. Control medium (10 mm CaCl_2_, 0.1 mm KCl, 2 mm Tris/MES, pH 5.8) was added at 35 s (black triangle). No significant difference between genotypes was found. (B) Response of Col-0 and *dorn1-1* to 0.1 mm ATP. (C) Response to 1 mm ATP. (D) Response to 1 mm ADP. (E) Total [Ca^2+^]_cyt_ mobilized (estimated as the area under the curve, after subtraction of mean pre-stimulus baseline) in response to control medium, 0.1 or 1 mm ATP, 1 mm ADP. *n* = 9–15 roots per genotype and treatment in three independent trials. Analysis of variance (ANOVA) with *post-hoc* Tukey test was used to assess statistical differences. Significance levels (*P*-values) in B–D: *** (<0.001); D: different lower-case letters indicate *P* < 0.05.

No studies to date have tested whether *dorn1* mutants can sustain eATP- or eADP-induced [Ca^2+^]_cyt_ elevation in their roots. Figure 2A shows that single excised roots of Col-0 and *dorn1-1* did not differ in their baseline [Ca^2+^]_cyt_ and were indistinguishable in their response to control solution. Addition of 0.1 mm ATP caused an initial touch response in both genotypes but only Col-0 then sustained the typical root biphasic [Ca^2+^]_cyt_ response after the initial touch response (Matthus *et al.*, 2019) (Fig. 2B). It is interesting to note that a biphasic response to eATP was sustained by Col-0 in the much simplified assay solution used here (10 mm CaCl_2_, 0.1 mm KCl; Laohavisit *et al.*, 2013) compared to the nutrient solution used by Matthus *et al.* (2019). This suggests a robust signalling system that remains unperturbed by environmental changes. The first and second peak [Ca^2+^]_cyt_ elevations of Col-0 were significantly greater than the [Ca^2+^]_cyt_ of *dorn1-1* at the equivalent time point (Fig. 2B) and the total [Ca^2+^]_cyt_ mobilized by eATP was also significantly greater in Col-0 (Fig. 2E). Addition of 1 mm ATP to Col-0 gave a less distinct biphasic [Ca^2+^]_cyt_ increase compared to 0.1 mm (in agreement with an earlier study by Demidchik *et al.*, 2003*a*) but this was still significantly greater than *dorn1-1* (Fig. 2C, E). The Col-0 response to 1 mm ADP was also biphasic and significantly greater than *dorn1-1* (Fig. 2D, E). Critically, while Col-0 [Ca^2+^]_cyt_ responses to purine nucleotides were all significantly higher than to control solution, those of *dorn1-1* were not (Fig. 2E) indicating that at this level of resolution (and in contrast to leaves; Fig. 1) the DORN1 receptor is essential.

### DORN1 abundance declines as root cells mature but is evident in trichoblasts

**Fig. 3.**
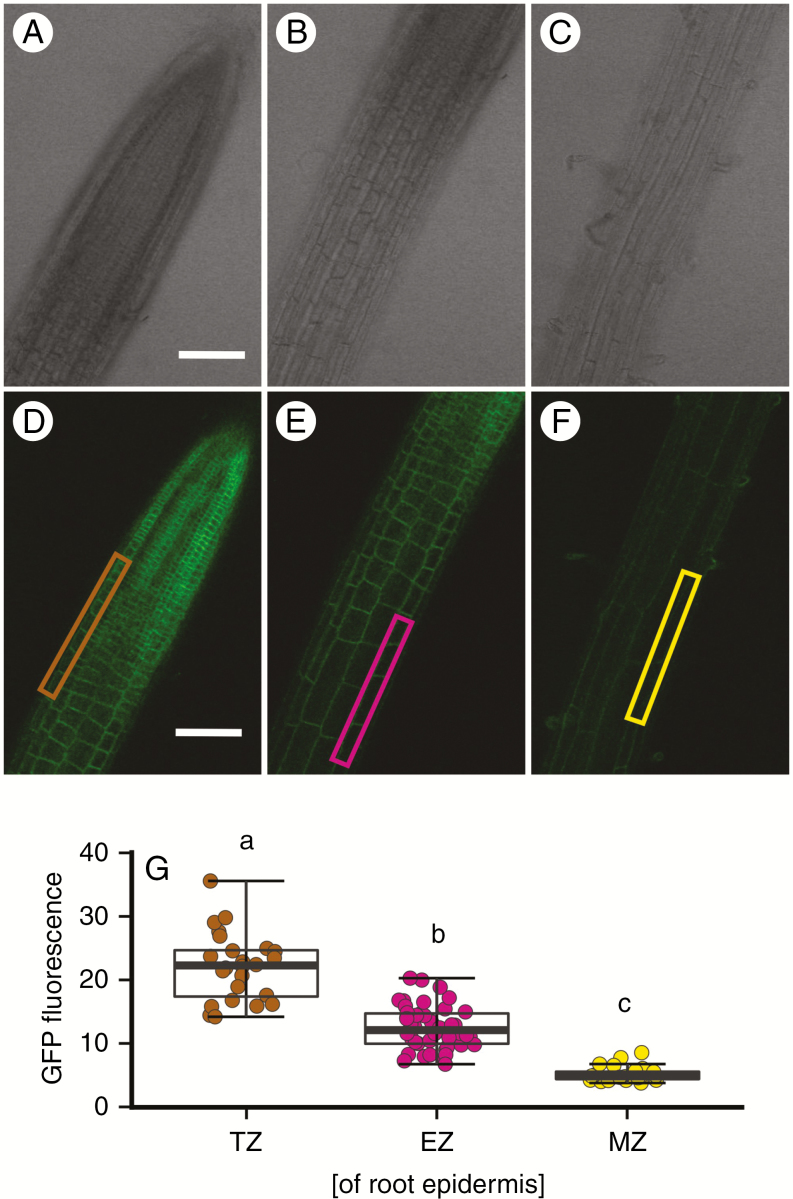
DORN1 abundance declines as primary root cells mature. (A–F) Confocal microscopy images of the different zones of 14-d-old *Arabidopsis* root expressing a DORN1-GFP fusion from the native promoter. Epidermis in the transition zone (TZ) is marked by brown rectangle, elongation zone (EZ) by pink, and mature zone (MZ) by yellow. Scale bar = 100 μm for all. (G) Background-subtracted DORN1-GFP fluorescence of epidermis in the transition zone (TZ, brown), elongation zone (EZ, pink) and mature zone (MZ, yellow) of 14-d-old *Arabidopsis* roots. GFP-signal was measured from the plasma membrane of epidermal cells and normalized to cells not expressing GFP; *n* = 24 (TZ/MZ) and *n* = 44 (EZ) from six individual plants. Analysis of variance (ANOVA) with *post-hoc* Tukey test was used to assess statistical differences, and different letters indicate significant difference (*P* < 0.001).

A GFP study previously confirmed DORN1’s plasma membrane localization (Choi *et al.*, 2014*a*). The same construct was used here. Abundance of DORN1 in the root epidermis declined as the cells matured from the transition zone (Fig. 3A, D, G), through to the elongation zone (Fig. 3B, E, G) and mature zone (Fig. 3C, F, G). Abundance was greatest in the first apical millimetre of root, which corresponds to the region that supports the apical and initial [Ca^2+^]_cyt_ increase by eATP (Matthus *et al.*, 2019). Of additional note is the appearance of DORN1 in the trichoblasts of the mature zone epidermis (Fig. 3F), which has not been reported previously.

### eATP may generate a [Ca^2+^]_cyt_ wave in the root

**Fig. 4.**
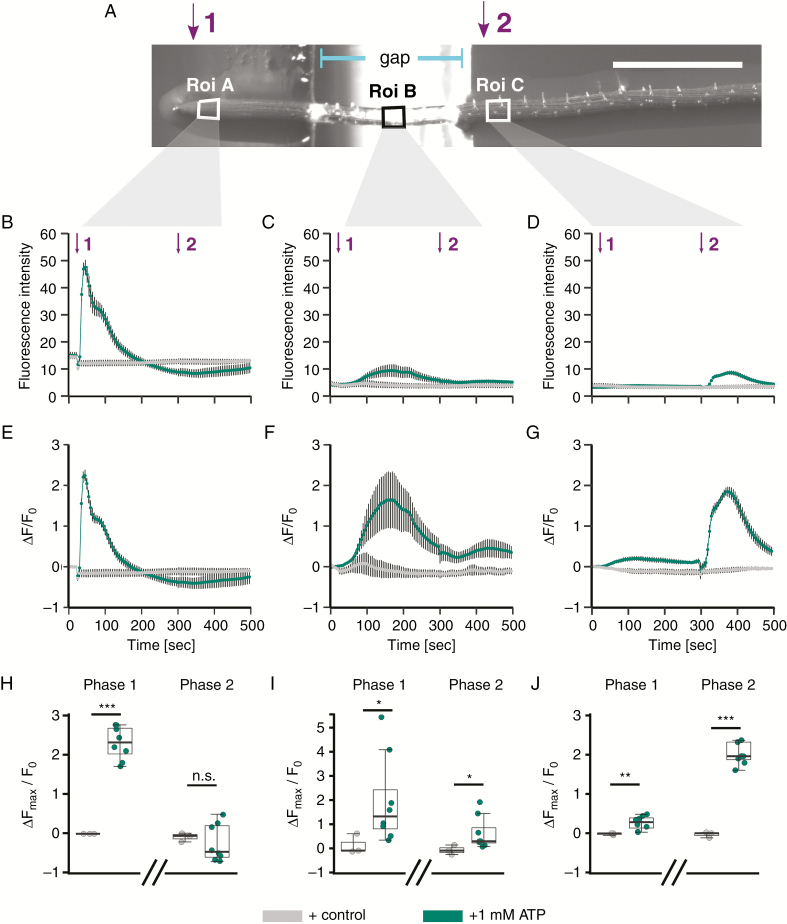
Localized ATP application causes spatially distinct [Ca^2+^]_cyt_ elevation in the root. (A) A 10-d-old *Arabidopsis* Col-0 seedling (expressing cytosolic GCaMP3; Vincent *et al.*, 2017) was placed across a gap in the growth medium agar; scale bar: 1 mm. Twenty seconds after the start of image acquisition, 3 µL of control or 1 mm ATP solution was applied to the root tip (indicated by purple ‘1’), and at 295 s to the mature zone (indicated by purple ‘2’), and then imaged for in total 495 s. Regions of interest used for analysis (‘Roi’) are annotated with white boxes. (B–D) Mean ± s.e.m. GFP fluorescence intensity, background-subtracted. (E–G) Normalized mean ± s.e.m. GFP fluorescence (Δ*F*/*F*_0_; Vincent *et al.*, 2017). (H–J) Extracted normalized fluorescence maxima (Δ*F*_max_/*F*_0_) for ‘Phase 1’ (20–295 s) and ‘Phase 2’ (300–495 s). Data shown in (B, E, H) represent Roi A; (C, F, I) represent Roi B; and (D, G, J) represent Roi C. Data are from three independent trials, with *n* = 3 individual roots for control treatments and *n* = 6–9 individual roots per ATP treatment. Significance levels (*P*-values, Welch’s two sample *t*-test) in H–J: *** (<0.001), n.s. (>0.05). Assay medium as in Fig. 1A.

Superfusion of a root with eATP causes apical and then sub-apical [Ca^2+^]_cyt_ responses that have the appearance of a Ca^2+^ wave (noted by, for example, Rincón-Zachary *et al.*, 2010; Loro *et al.*, 2016; Matthus *et al.*, 2019). However, the sub-apical [Ca^2+^]_cyt_ increases could simply be the result of direct cellular responses to eATP that are delayed in time rather than a consequence of the initial apical increase. Hence, it is unknown if eATP induces a Ca^2+^ wave which propagates away from locally treated areas. Here, a single root expressing cytosolic GCaMP3 (Vincent *et al.*, 2017) was placed over a gap in the underlying agar medium. The gap started after approximately the first millimetre of the root apex, so that it began after the region supporting the first [Ca^2+^]_cyt_ increase in response to eATP noted by Matthus *et al.* (2019) and after the greatest abundance of DORN1 (Fig. 3). eATP or control solution was applied sequentially to the root apex and then the mature region (Fig. 4A); the gap in the agar prevented capillarity-driven ATP movement between test regions (judged by previously testing fluorescein movement).

Regions of interest (‘Roi’) A, B and C were set for quantification of the GCaMP3 signal and corresponded to the apex, the region over the air gap and the mature zone respectively (Fig. 4A). Application of control solution did not lead to a fluorescence increase (Fig. 4B–J). Applying eATP first to the root apex (‘Phase 1’) revealed an immediate, largely monophasic fluorescence increase in the apex that recovered within 180 s (Roi A; Fig. 4B, E, H). This resembled the apical [Ca^2+^]_cyt_ increase reported by Tanaka *et al.* (2010) and Matthus *et al.* (2019) using YC3.6. Lower but significant transient increases were later also detected in Roi B (over the air gap) and Roi C (mature zone) (Fig. 4C–J). This indicated that local application of eATP treatment to the apical root tip triggered a [Ca^2+^]_cyt_ increase in tissue that did *not* come in contact with the treatment, travelling ~0.3–0.5 mm of untreated tissue before being detected in Roi C. A second eATP addition to the mature zone (‘Phase 2’) evoked a significant fluorescence increase there (Roi C; Fig. 4D, G, J) that did not evoke an increase at the apex (Fig. 4A, E, H).

**Fig. 5.**
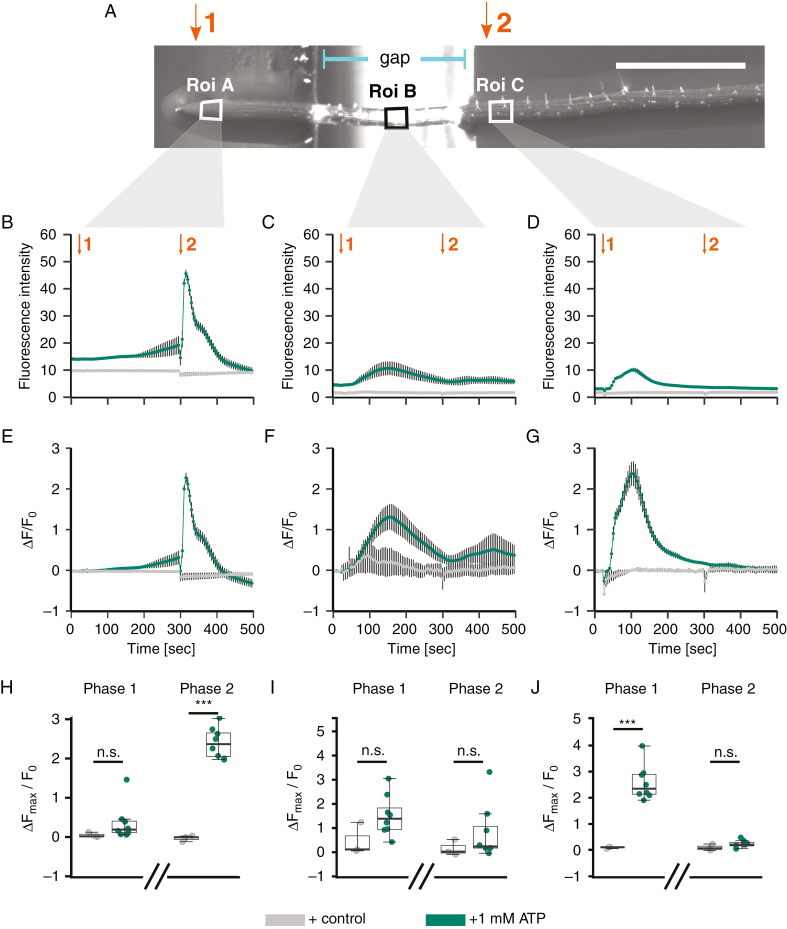
Localized ATP application to the mature zone causes spatially distinct [Ca^2+^]_cyt_ elevation in the root. (A) A 10-d-old *Arabidopsis* Col-0 seedling (expressing cytosolic GCaMP3; Vincent *et al.*, 2017) was placed across a gap in the growth medium agar; scale bar: 1 mm. Twenty seconds after the start of image acquisition, 3 µL of control or 1 mm ATP solution was applied to the mature zone (indicated by orange ‘1’), and at 295 s to the root tip (indicated by orange ‘2’), and then imaged for in total 495 s. Regions of interest used for analysis (‘Roi’) are annotated with white boxes. (B–D) Mean ± s.e.m. GFP fluorescence intensity, background-subtracted. (E–G) Normalized mean ± s.e.m. GFP fluorescence (Δ*F*/*F*_0_; Vincent *et al.*, 2017). (H–J) Extracted normalized fluorescence maxima (Δ*F*_max_/*F*_0_) for ‘Phase 1’ (20–295 s) and ‘Phase 2’ (300–495 s). Data shown in (B, E, H) represent Roi A; (C, F, I) represent Roi B; and (D, G, J) represent Roi C. Data are from three independent trials, with *n* = 3 individual roots for control treatments and *n* = 6–9 individual roots per ATP treatment. Significance levels (*P*-values, Welch’s two-sample *t*-test) in H–J: *** (<0.001), n.s. (>0.05). Assay medium as in Fig. 1A.

In a separate set of experiments, eATP was first added to the mature zone of the root (‘Phase 1’, Roi C) followed by application to the apex (‘Phase 2’, Roi A; Fig. 5A–G). Application to the mature zone caused a significant increase in [Ca^2+^]_cyt_ there compared to control solution (Fig. 5G, J) but although [Ca^2+^]_cyt_ increased in the root above the air gap (Roi B), it was not significant (Fig. 5F, I). The apex (Roi A) did not respond significantly when eATP was added to the mature zone. This confirms the previous result (Fig. 4E, H) that addition of eATP to the mature zone fails to evoke an apical response. When eATP was subsequently added to the apex (‘Phase 2’, Roi A), a significant [Ca^2+^]_cyt_ elevation occurred there. This showed that this region was competent to respond to a local stimulus (Fig. 5E, H) and perhaps was not in a refractory period that prevented it from responding to the eATP that had previously been added to the mature zone. Moreover, in contrast to the previous experiment in which eATP was added to the apex first (Fig. 4C–J), in this case addition of eATP to the apex *after* its addition to the mature zone did *not* cause significant [Ca^2+^]_cyt_ elevation in Roi B over the air gap or in the Roi C mature zone (Fig. 5E–J). Perhaps these zones were in a refractory period that prevented their response.

## DISCUSSION

### Leaf and root [Ca^2+^]_cyt_ signatures differ

The findings presented here clearly show that Arabidopsis leaf and root eATP-induced [Ca^2+^]_cyt_ signatures differ in their time courses and amplitude. Seedling responses would be an average of these. The leaf [Ca^2+^]_cyt_ response qualitatively resembled that reported for eATP-treated *Nicotiana benthamiana* leaf discs (DeFalco *et al.*, 2017). Furthermore, while the root signature appears to be entirely dependent on DORN1, other receptors may be operating in the leaf. This may be of use in the identification of other receptors and also in further studies on the possible roles of DORN1 in leaf purino-signalling. Certainly, the results here support the premise that eATP could affect response to cadmium, PSII activity and leaf jasmonate-dependent transcription through a [Ca^2+^]_cyt_ increase (Feng *et al.*, 2015; Hou *et al*., 2017, 2018). More detailed, higher resolution studies on cell-specific [Ca^2+^]_cyt_ responses are now needed for leaves and roots, such as those performed with YC3.6 on root responses to auxin (Shi *et al.*, 2015).

### An eATP-induced root wave?

So far, exogenous ATP has been applied by superfusion of roots or their total immersion. This does not allow us to distinguish between local or systemic effects of eATP perception. For example, locally applied salt treatment of Arabidopsis roots led to increases in [Ca^2+^]_cyt_, which propagated away and into areas of the plant that had not been in direct contact with the salt treatment, termed a ‘Ca^2+^ wave’ (Choi *et al.*, 2014*c*). Wave propagation involved both RBOHD and TPC1 (Evans *et al.*, 2016). A resultant transcriptional response was evident in the leaves. The same study found no such signal propagation upon mechanical stimulation, H_2_O_2_ or cold treatment, and did not test for the response to eATP (Choi *et al.*, 2014*c*). The results in Fig. 4 show that application of eATP to the root apex results in a sub-apical [Ca^2+^]_cyt_ elevation, consistent with a wave. However, the results in Fig. 5 show that this fails if the sub-apical region has already responded to a direct application of eATP, suggesting that there is a refractory period for the sub-apical response to apical stimulation. Furthermore, apical and mature region responses to eATP can be generated independently of one another and the mature region can still respond even though DORN1 abundance is lower there (Fig. 3). The response of the mature region to direct application of eATP may implicate the operation of another eATP sensing mechanism. The loss of DORN1 as the epidermis matures could also help to explain why eATP- and eADP-dependent net fluxes of Ca^2+^ and K^+^ also decline as the epidermis ages (Demidchik *et al.*, 2011). However, it should be noted that receptor abundance may not positively correlate with the magnitude of the response; much would depend on the levels of signal amplification and positive feedback downstream of the receptor. The results in Figures 4 and 5 suggest strongly that a reverse Ca^2+^ wave (sub-apical to apical) cannot be generated. Whether eATP application to the apex results in a systemic transcriptional response now needs to be examined.

### Placing DORN1 in the context of root ROS

Activation of RBOHs could be directly by DORN1-dependent phosphorylation and/or through their cytosolic EF hands (Takeda *et al.*, 2008) responding to an initial [Ca^2+^]_cyt_ elevation caused by DORN1-dependent plasma membrane Ca^2+^ channels. The latter would be consistent with the inhibitory effects of Gd^3+^ on eATP-induced ROS accumulation (Demidchik *et al.*, 2009). RBOHs would produce an extracellular superoxide anion that could readily be converted to H_2_O_2_ and from that to hydroxyl radicals (Richards *et al.*, 2015). From the guard cell paradigm, H_2_O_2_ could then enter into the cytosol through aquaporins (Rodrigues *et al.*, 2017) to be detected as part of the intracellular ROS accumulation. Both of those ROS are known to activate Arabidopsis root epidermal plasma membrane Ca^2+^ channels (Demidchik *et al*., 2003*b*, 2007; Foreman *et al.*, 2003). This would place some part of the plasma membrane Ca^2+^ influx *downstream* of RBOH activation in the eATP cascade, potentially acting to amplify the initial [Ca^2+^]_cyt_ elevation. This would be consistent with the inhibition of the eATP-activated HACC by the reductant dithiothreitol in mature epidermal plasma membrane (Demidchik *et al.*, 2009). As emerging root hairs are capable of ROS- and [Ca^2+^]_cyt_-dependent growth (Foreman *et al.*, 2003), the finding that DORN1 is present in trichoblasts suggests that DORN1 could be involved in root hair elongation through ROS- and [Ca^2+^]_cyt_-signalling.

### Plasma membrane calcium channel candidates

The finding that DORN1 can underpin both purine nucleotide-induced [Ca^2+^]_cyt_ increases in roots and leaves justifies this receptor as a logical starting point in the search for the proteins mediating plasma membrane Ca^2+^ fluxes. Direct phosphorylation of plasma membrane Ca^2+^ channels by DORN1 is a distinct possibility but, with channels likely to have a low copy number, approaches such as pull-down assays may not be fruitful in their identification. Publicly available protein–protein interaction data (MIND database; Jones *et al.*, 2014) revealed few interaction partners of DORN1, as only one uncharacterized leucine-rich repeat receptor kinase (At3g02880) was found to interact reliably. However, Chen *et al.* (2017) reported that in total 23 peptides were phosphorylated by DORN1 upon eATP perception, one of which was the RBOHD NADPH oxidase (the remaining 22 peptides were not further identified).

By extension of the leaf wound response, the root plasma membrane Ca^2+^ influx channels underpinning what may be a Ca^2+^ wave in the root could include GLR3.3 and 3.6 (Vincent *et al.*, 2017). Members of the cyclic nucleotide-gated channel (CNGC) family could also be involved, although so far Arabidopsis CNGC14 has been reported not to be involved in lateral root cap eATP-induced [Ca^2+^]_cyt_ elevation (Shi *et al.*, 2015). Annexins could also participate as channel regulators or unconventional ROS-activated channels and have been proposed to operate downstream of RBOHD (Laohavisit and Davies, 2011; Laohavisit *et al.*, 2012; Zandalinas *et al.*, 2019). Recently, Arabidopsis Annexin4 expressed in HEK cells was found to support [Ca^2+^]_cyt_ elevation in response to eATP (Ma *et al.*, 2019) but the operation of this annexin *in planta* in eATP signalling was not reported. Work needs to extend beyond Arabidopsis, as the legume root hair plasma membrane Ca^2+^ channels implicated in eATP/ADP-related symbiotic signalling remain to be discovered. Candidate root hair channel genes have been identified in *Medicago* and patch clamping the root hair apical plasma membrane of this legume is now firmly established (L. Wang *et al.*, 2019*b*) as a platform for such studies. Genes encoding the *Nicotiana tabacum* pollen plasma membrane eATP-activated channels also require identification (Wu *et al.*, 2018).

## CONCLUSIONS

Overall, DORN1 still provides an important experimental gateway into the further dissection of purine–calcium signalling in roots and leaves. However, comparisons with animal purino-signalling and some plant studies (including this) suggest that it is unlikely that DORN1 is the only purine nucleotide receptor in Arabidopsis. Other receptors may lie outside the lectin kinase family and may not even rely on the presence of an ATP-binding pocket. Novel ATP-binding sites have been reported for Arabidopsis peptides using acyl-ATP probes (Villamor *et al.*, 2013). Future studies also need to consider as yet unexplored areas of the plant. For example, as mechanical stress can result in release of ATP, does this occur during development of the shoot apical meristem? Does this link to its mechanically induced [Ca^2+^]_cyt_ increase that is mediated by Ca^2+^ influx (Li *et al.*, 2019)? While major breakthroughs on eATP signalling have come from Arabidopsis, it remains imperative that other plants (particularly monocot crops) continue to be studied. Apart from Arabidopsis, only tobacco has been reported to sustain an eATP-induced [Ca^2+^]_cyt_ signature (DeFalco *et al.*, 2017). [Ca^2+^]_cyt_ indicators have been introduced into *Medicago* and rice (Behera *et al.*, 2015; Kelner *et al.*, 2018) but as yet there are no reports of the effect of eATP. At present, another potential eATP receptor has been identified in *Camelina sativa* as an orthologue of DORN1 (csLecRK-I.9; Li *et al.*, 2016), but this is another brassica. Are there other DORNs?

## FUNDING

This work was supported by: UK Biotechnology and Biological Sciences Research Council (BBSRC) (BB/J014540/1); the framework of the 3^rd^ call of the ERA-NET for Coordinating Action in Plant Sciences, with funding from the BBSRC (BB/S004637/1), US National Science Foundation (grant 1826803) and the ANR; Jiangsu Normal University; Science and Engineering Research Board (India); University of Cambridge Commonwealth, European and International Trust; University of Cambridge Broodbank Trust; and Yayasan DayaDiri. Research reported from the Stacey laboratory was supported by the National Institute of General Medical Sciences of the National Institutes of Health (grant no. R01GM121445), as well as by the Next-Generation BioGreen 21 Program Systems and Synthetic Agrobiotech Center, Rural Development Administration, Republic of Korea www.rda.go.kr/foreign/ten/index.jsp (grant no. PJ01116604 to G.S.).
